# An application of extreme value theory to the management of a hydroelectric dam

**DOI:** 10.1186/s40064-016-1719-2

**Published:** 2016-01-29

**Authors:** Richard Minkah

**Affiliations:** Department of Statistics, University of Ghana, Accra, Ghana

**Keywords:** Extreme value theory, Generalized Pareto distribution, Probability weighted moments, Quantile estimation

## Abstract

Assessing the probability of very low or high water levels is an important issue in the management of hydroelectric dams. In the case of the Akosombo dam, very low and high water levels result in load shedding of electrical power and flooding in communities downstream respectively. In this paper, we use extreme value theory to estimate the probability and return period of very low water levels that can result in load shedding or a complete shutdown of the dam’s operations. In addition, we assess the probability and return period of high water levels near the height of the dam and beyond. This provides a framework for a possible extension of the dam to sustain the generation of electrical power and reduce the frequency of spillage that causes flooding in communities downstream. The results show that an extension of the dam can reduce the probability and prolong the return period of a flood. In addition, we found a negligible probability of a complete shutdown of the dam due to inadequate water level.

## Background

In the management of a hydroelectric dam, two events that can have devastating impact on the operations of the dam are very low and high water levels. The former has the tendency to cause a partial shutdown of the operations of the dam and in an extreme case a complete shutdown. On the other hand, the latter can cause flooding due to the spillage of excess water or dam failure in the worst-case scenario. Either way, the impact can be catastrophic with regard to power supply, environment, lives and properties. Therefore, modelling and estimating the frequency of these events remain an important issue for engineers and managers of dams. In this paper, extreme value theory (EVT) is used as a basis for the statistical analysis of very low and high water levels that can have adverse effect on the operations of the Akosombo hydroelectric dam.

EVT is a branch of statistics that deals with the statistical techniques for modelling and estimation of rare events. Unlike most traditional statistical analyses that deal with the center of the underlying distribution, EVT enables us to restrict attention to the behaviour of the tails of the distribution function. Thus, instead of measures of central tendencies such as mean, median and mode, the focus is on the examination of extreme (very small or very large) observations.

Fisher and Tippett (1928) laid the foundations for EVT for modelling and quantifying phenomena where events are rare and hence less or no data is available. Gnedenko (1943) unified and formalized the ideas of Fisher and Tippet into the fundamental assumption in EVT known as the extreme value condition. Gumbel (1958) was the first to give a statistical application of the theory to estimate extremes and the Gumbel distribution was named after him. Beirlant et al. (2004) reports that the theoretical aspects of EVT have its turning point from the doctoral dissertation by de Haan (1970) gave comprehensive properties of the sample extremes in a way that compares to the central limit theorem for the sample mean. Since then, interest in the field has been growing steadily and the main thematic research areas have centered on the following: construction of estimators for the extreme value index (EVI); threshold selection; estimation of large quantiles; and reduced bias estimators. In addition, the applicable areas of EVT includes insurance (Embrechts et al. 1997), finance (Embrechts et al. 1997; Gilli and Këllezi 2006), environmental science (Eastoe and Tawn 2009; Katz 2010), sport science (Einmahl and Magnus 2008; Henriques-Rodrigues et al. 2011), metallurgy (Beirlant et al. 2004), earth sciences (Dargahi-Noubary 1986; Pisarenko and Sornette 2003) among others. Moreover, EVT has been used to determine the safe heights for sea dikes in the Netherlands (de Haan 1990).

The construction of the Akosombo dam on the Volta river started in 1961 and was commissioned into operation in January, 1965. The dam is the largest hydroelectric dam in Ghana and provides electricity to Ghana and other neighbouring countries. It is also the largest man-made lake in the world with regard to surface area at 8502 km^2^. Besides rain water, the lake has its major inflow source from the black Volta, the white Volta and the Oti river. The dam has six units of turbine-generators with a combined generating capacity of 1020 MW. This accounts for over 40 % of the entire electricity generation mix of Ghana (VRA 2013). In addition, there are a number of spill ways for spilling excess water.

The dam’s operation depends on the level of head water which must be between a minimum and maximum operating level of 240 and 278 feet (ft) respectively. Some of the turbines are shutdown during periods with water levels below 240 ft and this usually result in load shedding of electricity (i.e. a planned electrical power shutdown in parts of the country to prevent the collapse of the entire power system). In addition, the inlet surface of the dam stands at 226 ft: this is the “critical” level above which water can run through the penstocks to generate electrical power. Thus, the generation of electricity from the dam will come to a complete halt for water levels below 226 ft. On the other hand, during spells of high water levels close to 278 ft, the excess water is spilled to avoid overflow or dam failure. The spillage usually causes flooding in communities downstream with its attending destruction to lives and properties.

Taking all these into consideration, EVT offers a solid mathematical foundation to determine extreme cases (very low or high) of water levels in the dam. In this regard, the focus of the paper is to use EVT to analyse the water levels under the present working conditions of the dam to determine:

1. if the water level can fall below the critical level of 226 ft;
2. how high a proposed extension should be such that the probability of a flood in a given year is $p =1/100~$ [i.e. 100-year (1200-month) return level of a flood];
3. and for any given height (in ft), the probability that the water level will fall below or rise above it.

The rest of the paper is organized into three sections. The “Extreme value theory” section provides an overview of EVT with emphasis on the peaks-over threshold (POT) method and the estimation of parameters of extreme events. In the “Extreme value analysis of water levels” section , the estimation techniques described in the previous section are used to analyse the data on the water levels of the Akosombo hydroelectric dam. Finally, the “Conclusions” section provides the conclusions drawn from the data analysis in “Extreme value analysis of water levels” section.

**Table 1:** Summary statistics of water levels

Statistic	Left tail	Right tail	Overall data
Minimum	234.96	235.48	234.96
1st quartile	247.05	249.74	248.52
Median	255.63	257.9	256.90
3rd quartile	264.56	266.77	265.71
Maximum	276.68	277.54	277.54
Standard deviation	10.45	10.41	10.46

## Extreme value theory

Consider a sequence of independent and identically distributed random variables ${\{X_1, X_2,\ldots, X_n\} }$ with distribution function *F*. Let the associated order statistics be given by $X_{1,n}\le X_{2,n} \le \cdots \le X_{n,n}.$ Suppose the variable of interest is the maximum,

$$\begin{aligned} X_{n,n}=\max {\{X_1, X_2,\ldots, X_n\}} \end{aligned}$$

or the minimum,

$$\begin{aligned} X_{1,n}=\min {\{X_1, X_2,\ldots, X_n\}}=-\max {\{-X_1, - X_2,\ldots, -X_n\}}, \end{aligned}$$

then, the distribution function of $X_{n,n}$ is related to the underlying distribution function *F* as

1 $$\begin{aligned} F_{X_{n,n}}(x)=F^n(x). \end{aligned}$$

However, *F* is usually unknown and hence in EVT, $F^n$ is approximated by limit distributions as $n \rightarrow \infty$. Fisher and Tippett (1928) and Gnedenko (1943) proved that a properly centered and normalised $X_{n,n},$ converges in distribution to a non-degenerate limit, which is necessarily an extreme value distribution. This is formally stated in Coles (2001, p. 46) as:

### **Theorem 1**

(Fisher–Tippet Theorem) *If there exist sequences*$a_n>0$*and*$b_n\in {\mathbb {R}}$*such that*

2 $$\begin{aligned} \lim \limits _{n\rightarrow \infty }P\bigg (\frac{X_{n,n}-b_n}{a_n}\bigg )\rightarrow G_\gamma (x), \end{aligned}$$

*where G is a non-degenerate function, then G belongs to one of the extreme value distributions given by*

(I) $$G_\gamma (x)=\exp {\left( -\exp {\left( -\frac{x-b}{a}\right) }\right) }, \quad x\in {\mathbb {R}} \; (\gamma =\alpha =0)$$
(II) $$G_\gamma (x)=\left\{ \begin{array}{ll} 0, & \quad \text{ if } \; x \le b,\\ \exp \left( -\left( \frac{x-b}{a}\right) ^{-\alpha }\right) , & \quad \text{ if } \; x>b, \; \alpha >0 \; \left( \gamma =\frac{1}{\alpha }>0\right) . \end{array} \right.$$
(III) $$G_\gamma (x)= \left\{ \begin{array}{ll} \exp \left( -\left( -\frac{x-b}{a}\right) ^\alpha \right) , & \quad \text{ if }\; x<b, \; \alpha >0 \;\left( \gamma =-\frac{1}{\alpha }<0\right) \\ 1, & \quad \text{ if } \; x \ge b. \end{array} \right.$$

*for all*$a>0$* and*$b\in {\mathbb {R}}.$

The class of the limiting distributions (I), (II) and (III) are referred to as Gumbel, Pareto and Weibull types of extreme value distribution respectively. Jenkinson (1955) obtained a representation for the three classes termed as the generalised extreme value (GEV) distribution. The distribution function of the GEV is given by

3 $$\begin{aligned} G_\gamma (x)=\left\{ \begin{array}{ll} \exp \left (-\left (1+\gamma \frac{x-\mu }{\sigma }\right )^{-1/\gamma }\right ), &\quad 1+\gamma \frac{x-\mu }{\sigma }>0,~ \gamma \ne 0;\\ \exp \left (-\exp \left (\frac{x-\mu }{\sigma }\right )\right ), &\quad x\in {\mathbb {R}}, ~\gamma =0.\end{array} \right. \end{aligned}$$

where $\gamma \in {\mathbb {R}},~\mu \in {\mathbb {R}}$ and $\sigma >0$ are the shape, location and scale parameters respectively. In the literature, $\gamma$ is usually referred to as the extreme value index (EVI) or tail index. It determines the tail heaviness of the extreme value distributions. The cases $\gamma =0, ~ \gamma >0 ~\text{ and }~\gamma <0$ correspond to the Gumbel, Pareto and the Weibull domains of attraction respectively. The distribution function in the Pareto domain are heavy-tailed distributions; the Weibull domain contains short-tailed (bounded) distributions; and the Gumbel domain contains light-tailed distributions. From this result, Gumbel (1958) proposed estimating $\gamma$ by fitting the distribution function, *G*,  to sample maxima. The parameters $\gamma ,~ \mu$ and $\sigma$ of the GEV distribution can be estimated with the probability-weighted moments (PWM) (Hosking et al. 1985), maximum likelihood method (Prescott and Walden 1980; Smith 1985), and Bayesian estimation (Lye et al. 1993). However, this approach is known to waste data.

**Table 2:** Estimates of exceedance/deceedance probabilities and return periods

No.	Left tail			Right tail		
	Level of dam	Deceedance probability	Return period (in years)	Level of dam	Exceedance probability	Return period (in years)
1	231.00	3.41e−06	244	278.00	1.23e−02	7
2	232.00	1.94e−03	43	278.50	5.18e−03	16
3	233.00	7.17e−03	12	279.00	1.16e−03	52
4	234.00	2.05e−02	4	279.50	2.57e−04	324
5	235.00	4.93e−02	2	280.00	3.15e−06	26438

An alternative method that makes efficient use of the data is the peaks-over threshold (POT) method. The POT method focuses on fitting an appropriate parametric distribution to observations in a sample that exceed a sufficiently high threshold. Assuming that there are enough observations above the threshold, we look for an appropriate conditional distribution for these excesses or exceedances. Let $X=(X_1, \ldots , X_n)$ be a random sample with an underlying distribution *F* and $x^F=\sup \{x: F(x)<1\}$ be the right endpoint of *F*. In addition, let *u* denote the threshold value such that $u<x^F,$ and the distribution of the exceedances,

4 $$\begin{aligned} F_u(x)=P\left( X>u+x|X>u\right) =\frac{1-F(u+x)}{1-F(u)},\quad x\ge 0. \end{aligned}$$

The Pickands–Balkema–de Haan theorem describes how under some general conditions, the limiting distribution of the excesses is described by the generalized Pareto (GP) distribution (Balkema and de Haan 1974; Pickands 1975). The GP distribution is specified by

5 $$\begin{aligned} H_\gamma (x)=\left\{ \begin{array}{ll} 1-\left (1+\frac{\gamma (x-u)}{\sigma _u}\right )^{-1/\gamma },& \quad 1+\frac{\gamma (x-u)}{\sigma _u}\ge 0, \; x\ge u, \; \text{ if }\; \gamma \ne 0,\\ 1-\exp \left (-\frac{x-u}{\sigma _u}\right ), & \quad x\ge u, \; \text{ if }~ \gamma =0, \end{array}\right. \end{aligned}$$

where $\gamma$ and $\sigma _u$ are the shape and scale parameters respectively. Here, the shape parameter, $\gamma$, is the EVI or the tail index. The distribution belongs to the Pareto domain for $\gamma >0$, Gumbel domain for $\gamma =0$, and the Weibull domain for $\gamma <0.$ The Pickands–Balkema–de Haan theorem is stated as follows:

### **Theorem 2**

(Pickands–Balkema–de Haan Theorem) *Let F be a distribution function of X and the distribution of excesses *$Y=X-u$* over a threshold u denoted by *$F_u.~ F\in D(H_\gamma )$* if and only if*

6 $$\begin{aligned} \lim \limits _{u\rightarrow x^F}\left|F_u(y)-H_\gamma (y|\sigma _u)\right|=0, \end{aligned}$$

*where*$\gamma$*and*$\sigma _u$*are the shape and scale parameters of the GP distribution function H*.

The parameters of the GP distribution can be estimated with the probability-weighted moments (Hosking and Wallis 1987) and the maximum likelihood method (Smith 1984) among others.

An important consideration in the process of fitting a GP distribution is the choice of threshold, *u*. A high threshold results in few observations leading to large variation in estimators. On the other hand, a low threshold results in the inclusion of moderate observations leading to large bias. Therefore, a compromise has to be found between bias and variance. We refer the reader to Scarrott and MacDonald (2012) for a thorough review of existing methods in the literature for threshold selection.

### Parameter estimation of the GP distribution

In EVT, the most important parameters of interest include high/low quantiles (return levels), exceedance/deceedance probabilities, return periods and right/left endpoints of the distribution function, *F*. However, all the parameters of extreme events depend on the EVI. Thus, the EVI is of primordial importance and must be estimated before any meaningful extreme value analysis can be done.

Let $n_u$ be the number of observations in the sample $(X_1, \ldots , X_n)$ exceeding the threshold *u*,  and $Y_1, \ldots , Y_{n_u}$ be the excesses where $Y_j=X_i-u$ with $i=1,\ldots ,n$ and $j=1,\ldots , n_u.$ We know from Theorem 2 that the limiting distribution of the excesses is the GP distribution. In this paper, we estimate the parameters $\sigma _u$ and $\gamma$ of the GP distribution with the probability weighted moments (PWM) only. The PWM is known to perform better than the maximum likelihood estimators for small sample sizes and for some range of values of $\gamma$ (Hosking and Wallis 1987).

The PWM is a generalization of the method of moments with tail observations assigned more weights. For a random variable *X*,  the PWM is defined as

7 $$\begin{aligned} M_{p,r,s}=E\left( X^p\left( F(X)\right) ^r(1-F(X))^s\right) , \end{aligned}$$

for $p, r, s \in {\mathbb {R}}.$ Hosking and Wallis (1987) considered $M_{p,r,s}$ with $p=1, r=0$ and $s=0, 1,\ldots,$ giving

8 $$\begin{aligned} M_{1,0,s}=\frac{\sigma _u}{(s+1)(s+1-\gamma )}, \quad \gamma <1. \end{aligned}$$

Here, the parameter $M_{1,0,s}$ can be replaced by its empirical estimator

9 $$\begin{aligned} \hat{M}_{1,0,s}=\frac{1}{n_u}\sum _{j=1}^{n_u}\left( \prod _{i=1}^{s}\frac{n_u-j-i+1}{n_u-i}\right) Y_{j,n_u}. \end{aligned}$$

Substituting $M_{1,0,s}$ with the estimator in (9) and solving for $s=0$ and $s=1$ with respect to $\gamma$ and $\sigma _u$ yields the PWM estimator

10 $$\begin{aligned} \hat{\gamma }=2-\frac{\hat{M}_{1,0,0}}{\hat{M}_{1,0,0}-2\hat{M}_{1,0,1}} \end{aligned}$$

and

11 $$\begin{aligned} \hat{\sigma }_u=\frac{2\hat{M}_{1,0,0}\hat{M}_{1,0,1}}{\hat{M}_{1,0,0}-2\hat{M}_{1,0,1}}. \end{aligned}$$

for $\gamma$ and $\sigma _u$ respectively. The authors showed that the PWM estimators have asymptotic normality i.e.

12 $$\begin{aligned} \sqrt{n_u}\left( \left(\hat{\gamma },\hat{\sigma _u}\right)-(\gamma , \sigma _u)\right) \overset{d}{\longrightarrow } N(\varvec{0},{\mathbb {I}}^{-1}) \end{aligned}$$

for $n_u \rightarrow \infty ,$ where ${\mathbb {I}}^{-1}$ is the inverse of the Fisher information matrix. Therefore, for statistical inference, normal confidence intervals can be constructed for the parameters $\gamma$ and $\sigma _u.$ Let $\varvec{\theta } =(\gamma , ~\sigma _u),$ and $\hat{\varvec{\theta }}$ the PWM estimator of $\varvec{\theta }.$ The $100 (1-\alpha )\%$ normal confidence interval of $\varvec{\theta }$ is given by,

13 $$\begin{aligned} \hat{\varvec{\theta }} \pm \Phi ^{-1}{(1-\alpha /2)}\sqrt{\frac{v(\hat{\varvec{\theta }})}{m}}. \end{aligned}$$

Here $v(\hat{\varvec{\theta }})$ represents the diagonal elements in the variance-covariance matrix of the limiting normal distribution.

### Estimation of other parameters of extreme events

Having estimated the parameters of the GP distribution, other important parameters of extreme events i.e. exceedance/deceedance probabilities, quantiles (return levels) and return periods can be obtained. The $(1-p)$-th quantile, with $p\rightarrow 0$ is obtained by inverting (5),

14 $$\begin{aligned} Q_Y(1-p)=\left\{ \begin{array}{ll} \frac{\sigma _u}{\gamma }\left( p^{-\gamma }-1\right) ,&\quad \text{ if }~\gamma \ne 0,\\ \sigma _u\log p , &\quad \text{ if }~\gamma =0.\end{array} \right. \end{aligned}$$

Substituting $\gamma$ and $\sigma _u$ in (14) with the respective PWM estimators $\hat{\gamma }$ and $\hat{\sigma }_u$ result in the estimator for extreme quantiles.

The quantile estimation can also be expressed in terms of the underlying random variable *X*. From (4) and Theorem 2, we have

15 $$\begin{aligned} P\left( X>x|X>u\right) =\left( 1+\frac{\gamma (x-u)}{\sigma _u}\right) ^{-1/\gamma }. \end{aligned}$$

Therefore, it follows that

16 $$\begin{aligned} P(X>x)=\bar{F}(u)\left( 1+\frac{\gamma (x-u)}{\sigma _u}\right) ^{-1/\gamma }, \end{aligned}$$

where $\bar{F}=1-F$ is the survival function. Estimating $\bar{F}(u)$ by the proportion of exceedances in the sample, $n_u/n,$ and replacing the pair $(\gamma , \sigma _u)$ by the PWM estimator $(\hat{\gamma }, \hat{\sigma }_u)$ yields an estimator of the tail probability, $P(X>x),$ i.e.

17 $$\begin{aligned} \hat{\bar{F}}(x)=\frac{n_u}{n}\left( 1+\frac{\hat{\gamma } (x-u)}{\hat{\sigma }_u}\right) ^{-1/\hat{\gamma }}. \end{aligned}$$

The $(1-p)$-th quantile estimator of the underlying random variable *X* for the case $\gamma \ne 0$ can be obtained by solving for *x* in (17),

18 $$\begin{aligned} \hat{Q}_X(1-p)=u+\frac{\hat{\sigma }_u}{\hat{\gamma }}\left( \left( \frac{n_u}{np}\right) ^{\hat{\gamma }}-1\right) . \end{aligned}$$

In the case of $\gamma =0,$ similar arguments lead to the estimator of an extreme quantile,

19 $$\begin{aligned} \hat{Q}_X(1-p)=u+\hat{\sigma }_u\log \left( \frac{n_u}{np}\right) . \end{aligned}$$

In addition, if $\gamma <0,$ the right endpoint of the underlying distribution function *F* is obtained by taking the limit as $p\rightarrow 0$ in (18),

20 $$\begin{aligned} \hat{x}^F=u-\frac{\hat{\sigma }_u}{\hat{\gamma }}. \end{aligned}$$

Furthermore, the return period associated with a $(1-p)$-th extreme quantile is defined as

21 $$\begin{aligned} R_p=\frac{1}{p}. \end{aligned}$$

Confidence intervals for quantiles and exceedance probabilities can be constructed by using the limiting normal distribution (12) and the delta method (Coles 2001; Beirlant et al. 2004).

## Extreme value analysis of water levels

In this section, we present an extreme value analysis of the water levels of the Akosombo dam. Firstly, we describe the basic characteristics of the data and then fit the GP distribution to the data. Lastly, we estimate the other parameters of extreme events.

**Fig. 1:**
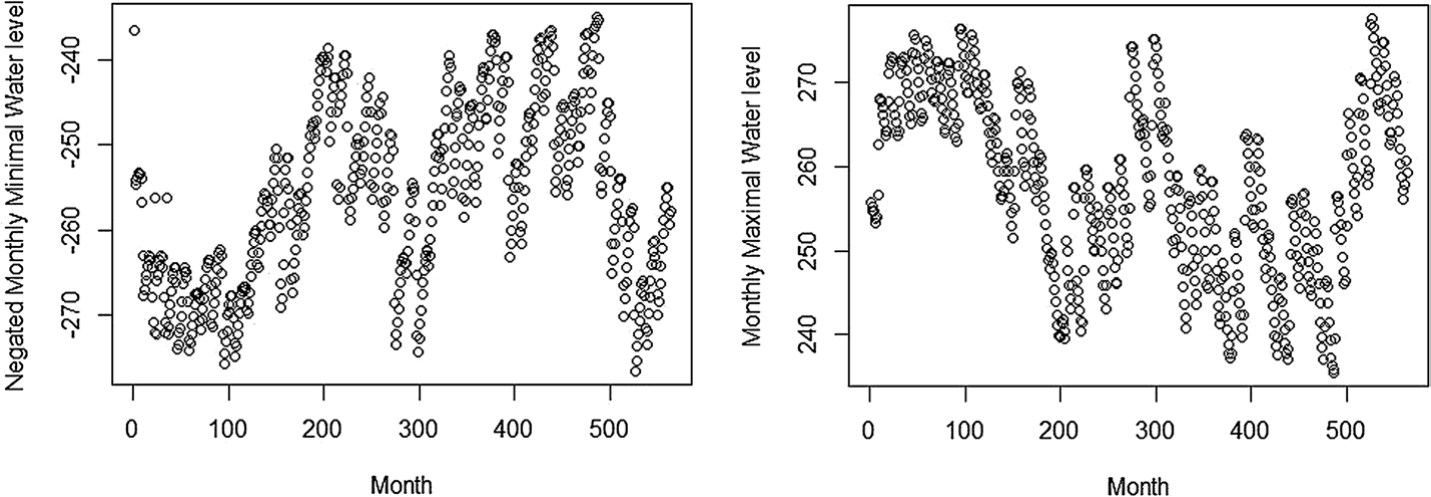
Plot of water levels: negated monthly minimal water levels, *left panel*; monthly maximal water levels, *right panel*

The data consists of 576 pairs of observations of monthly minimal and maximal water levels from the Akosombo dam between the periods January, 1966 and December, 2013. Figure 1 shows the monthly minimal and maximal water levels for the period under consideration. The monthly minimal and maximal water levels are used to study the left and the right tails of the underlying distribution of water levels respectively. In addition, we negated the monthly minimal water level values due to the duality between the distributions for maxima and minima as illustrated in the “Extreme value theory” section. Thus, both problems were considered as a maxima problem.

The data exhibit some clustering at extreme levels i.e. a month with high (low) water level is likely to be followed by another month with high (low) water level. Such dependence in the data calls into question the independence assumption underlying the GP distribution. Procedures for addressing the problem of dependent exceedances can be found in Leadbetter et al. (1983), Beirlant et al. (2004), and Embrechts et al. (1997). In addition, Coles (2001) provides a basic procedure to deal with dependent data called declustering. It involves blocking the observations into clusters and the cluster maxima are taken as the independent sample of maxima. Thus, the declustering procedure is used to filter the data so as to achieve a (near-) independent sample of maxima for the application of the POT method. However, only cluster maxima are used and this leads to a less optimum use of data. In our case, the declustering procedure resulted in between 5 and 20 exceedances depending on the number of clusters. However, ignoring the dependence in the data implies that we risk underestimating the return levels and return periods (see e.g. Beirlant et al. 2004; Coles 2001). Such a conservative approach is better in the context of managing a risky operation of a hydroelectric dam. In other words, it is prudent to plan towards shorter return periods of catastrophic events provided by the independent assumption. Therefore, we assume that the water levels are independent and apply the POT method in this study.

Table 1 shows the summary statistics of the monthly minimal and maximal water levels. We note that, several water levels recorded were below the minimum operating level of 240 ft but greater than the critical level of 226 ft. As a result, some of the turbines are temporally shutdown on numerous occasions leading to power cuts. However, there has not been a complete shutdown of the dam due to low water levels. On the other hand, the maximum water level recorded was 0.46 ft below the maximum operating level of the dam at 278 ft. When the water level inches towards 278 ft, the dam’s spill ways are opened to spill excess water in order to avoid an overflow or dam failure. The spillage causes flooding in the communities downstream and the most recent incident was October, 2010.

**Fig. 2:**
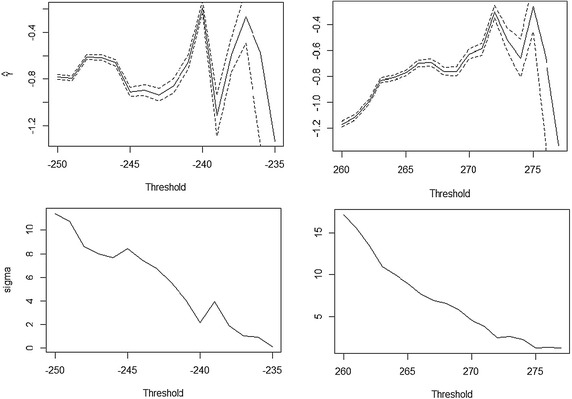
Parameter estimates of the GP distribution: negated monthly minimal water levels, *left panel*; monthly maximal water levels, *right panel*

**Fig. 3:**
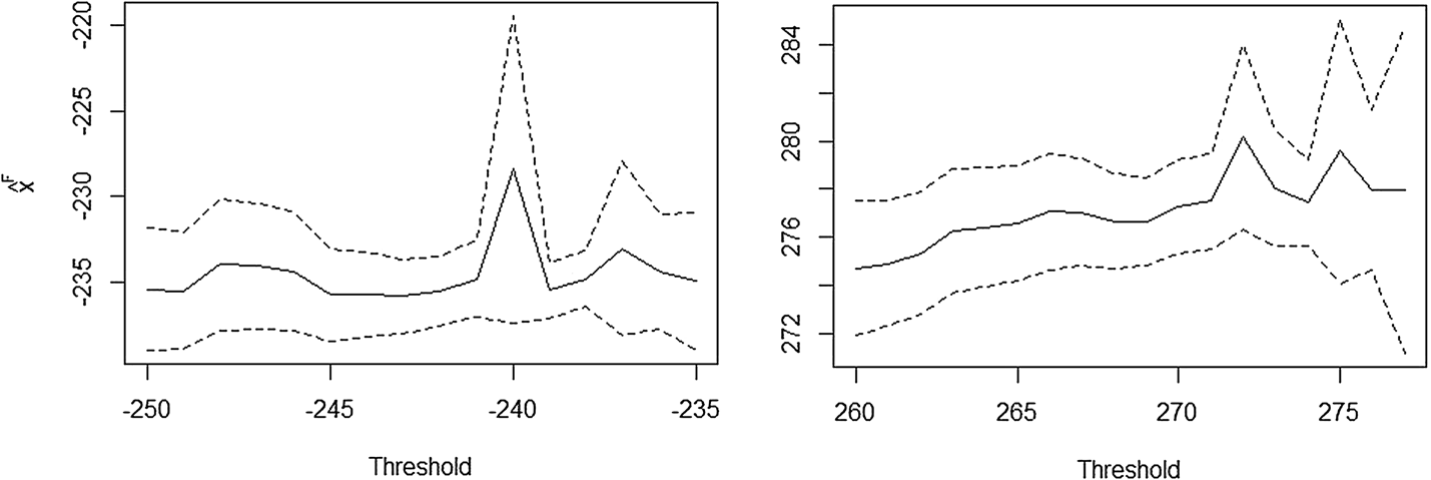
Estimates of the endpoints of underlying distribution of water levels: left endpoint, *left panel*; and right endpoint, *right panel*

The PWM estimates of $\gamma$ and $\sigma _u$ at various thresholds are shown in Fig. 2. The estimates were obtained from *R* package *evir* and the codes are available upon request from the author. All the estimates of $\gamma$ and its 95 % confidence interval band for both tails at each threshold value are negative. Thus, we conclude that both tails belong to the Weibull domain of attraction: the underlying distribution of the monthly minimal and maximal water levels are bounded on the left and right tails respectively. Using (20), the estimated left and right endpoints for the various thresholds are shown in the left and the right panels of Fig. 3 respectively. Since our interest is in assessing the exceedance probabilities and return periods of some selected levels of the dam, the criterion for selecting the thresholds was the ability to provide reasonable answers to the questions posed in the “Background” section.

Table 2 shows the return periods of very low and high water levels for selected levels of the dam resulting in shutdown of turbines and flood respectively. From this table, we make the following deductions to address the three questions in “Background” section respectively.

Firstly, we consider the left tail of the underlying distribution of water levels to provide an answer to question 1. In this case, the minimum operating level of 240 ft provides a natural threshold resulting in approximately 10 % deceedances of the monthly minimal water levels. The estimate of $\gamma$ = −0.187 and the 95 % confidence interval is [−0.240, −0.134]. The corresponding estimate of the left endpoint is 228.402 ft with a 95 % confidence interval, [219.431, 237.374] ft. Thus, the left endpoint estimate is greater than the critical level of 226 ft but the 95 % confidence interval estimate encloses this value. Therefore, we conclude that there is a negligible chance of a complete shutdown of the dam due to low water levels.

Secondly, with regard to the right tail, we selected a threshold value of 272 ft resulting in 56 monthly maximal exceedances. The estimate of $\gamma$ at this threshold equals −0.30 and the 95 % confidence interval is [−0.349, −0.252]. In addition, the right endpoint value at this threshold is 280.180 ft. The corresponding 95 % confidence interval for the right endpoint is [276.327, 284.036] ft. Since the right endpoint estimate at this threshold value is greater than the maximum operating level (i.e. 278 ft) of the dam, we can compute the exceedance probabilities and return periods beyond the maximum operating level. An increase of more than 1 ft of the dam’s maximum operating level result in a value surpassing the usual 100-year return period of a flood. Therefore, this affords engineers a scientific basis to consider an extension of the dam to reduce the occurrence of flooding and retain more water for the generation of electrical power.

Lastly, the results of the left panel show that the water level is expected to drop below 235 ft (i.e. 5 ft less than the minimum operating level) once every 2 years. As a result, some turbines are expected to be shutdown at least once in every 2 years due to inadequate water levels. Also, the 100-year return level in this case is between 231 and 232 ft. On other hand, the right panel shows the exceedance probabilities and the associated return periods for levels between 278 and 280 ft. The results show that an extension of the maximum operating level of the dam to 279 ft will increase the return period of a flood to approximately once in every 52 years. However, an additional 1 ft extension of the level of the dam increases dramatically the return period as the exceedance probability approaches zero.

**Fig. 4:**
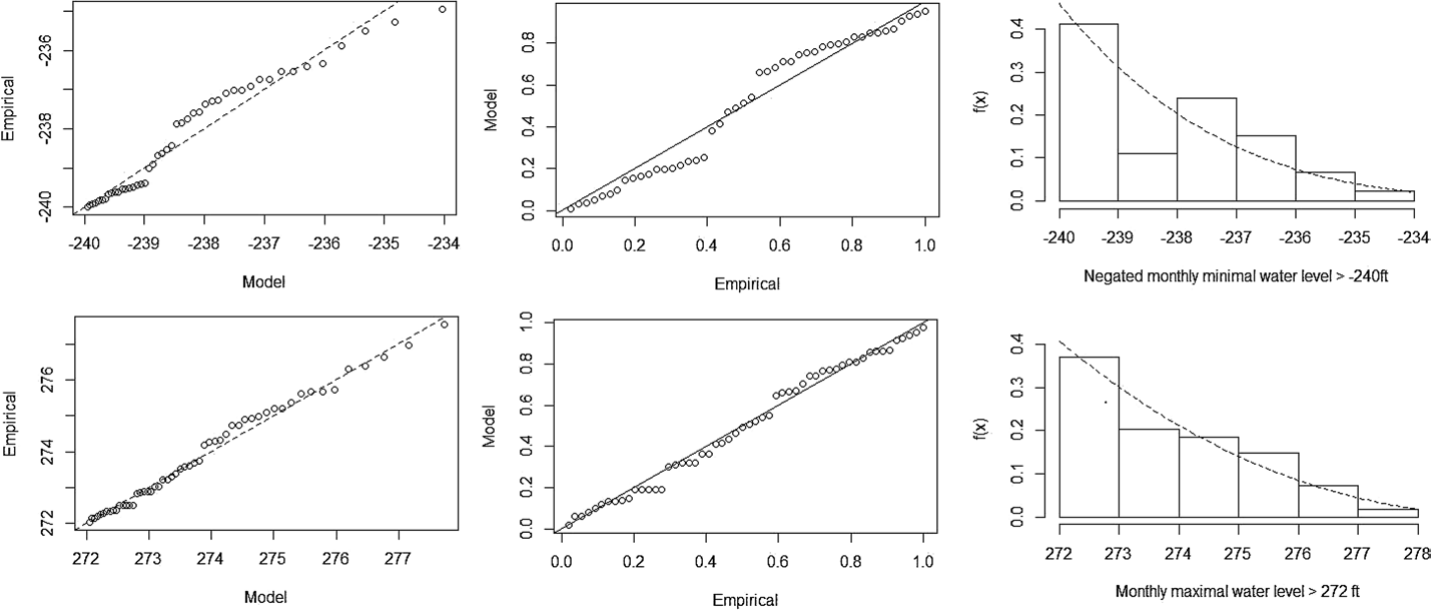
Diagnostic plots for GP fit to the Akosombo water level data. The *top panel* shows the plots for negated monthly minimal water levels; the *bottom panel* shows the plots for monthly maximal water levels. In addition, the *leftmost column* shows the QQ-plots; *middlemost column* shows the PP-plots; and the *rightmost column* shows the density estimates superimposed on the histogram of data

We now proceed to perform some diagnostic checks on the accuracy of the fitted GP distribution at the selected thresholds for the left and right tails of the distribution of water levels. Figure 4 presents the quantile–quantile (QQ) plot, probability–probability (PP) plot and the conditional histogram for water level with the fitted GP density superimposed. The QQ and PP plots exhibit a general linear trend. Also, the density plot seems consistent to the fitted histogram especially at the extreme tails. In general, we can conclude that the diagnostic plots show satisfactory support for the fitted GP distributions. In particular, the fit is better in the right tail of the distribution. This can also be seen from the confidence intervals for the parameters of the GP distribution and extreme events: the right tail have shorter interval lengths compared to the corresponding interval lengths on the left tail.

## Conclusions

We have shown that extreme value theory (EVT) and in particular the POT method offers a good statistical tool for the description of water levels of the Akosombo dam. It allows us to restrict attention to very low and high water levels. The former has implications for the smooth running of the dam to generate electricity; and the latter, the safety of the dam and its adjoining environments.

The results demonstrate that under the current working conditions of the dam, there is a negligible chance of a complete shutdown of the dam due to inadequate water level. Similarly, we provided a framework that gives engineers the basis to consider an extension of the maximum operating level of the dam to reduce spillage of excess water to once in every 100 years or beyond.

The present study implicitly makes the assumption of stationarity with respect to the influence of climatic conditions on the water levels of the dam. Some of these climatic conditions (e.g. rainfall and temperature) can be taken alongside other factors including volume of inflows and discharged water as covariates to improve estimation and statistical inference. However, some additional research is needed in the future to evaluate the relative merits of the inclusion of these covariates and our present study.

## References

[CR1] Balkema AA, de Haan L (1974). Residual life time at great age. Ann Probab.

[CR2] Beirlant J, Goegebeur Y, Segers J, Teugels J (2004). Statistics of extremes: theory and applications.

[CR3] Coles S (2001). An introduction to statistical modeling of extreme values.

[CR4] Dargahi-Noubary GR (1986). A method for predicting future large earthquakes using extreme order statistics. Phys Earth Planet Inter.

[CR5] de Haan L (1970) On regular variation and its application to the weak convergence of sample extremes. University of Amsterdam, Ph.D.

[CR6] de Haan L (1990). Fighting the archenemy with mathematics. Stat Neerl.

[CR7] Eastoe EF, Tawn JA (2009). Modelling non-stationary extremes with application to surface level ozone. J R Stat Soc: Ser C (Appl Stat).

[CR8] Einmahl JHJ, Magnus JR (2008). Records in athletics through extreme-value theory. J Am Stat Assoc.

[CR9] Embrechts P, Klüppelberg C, Mikosch T (1997). Modelling extremal events: for insurance and finance.

[CR10] Fisher RA, Tippett LHC (1928). On the estimation of the frequency distributions of the largest or smallest member of a sample. Proc Camb Philos Soc.

[CR11] Gilli M, Këllezi E (2006). An application of extreme value theory for measuring financial risk. Comput Econ.

[CR12] Gnedenko B (1943). Sur la distribution limite du terme Maximum d’une série aléatoire. Ann Math.

[CR13] Gumbel EJ (1958). Statistics of Extremes.

[CR14] 
Henriques-Rodrigues L, Gomes MI, Pestana D (2011). Statistics of extremes in athletics. REVSTAT.

[CR15] Hosking JRM, Wallis JR (1987). Parameter and quantile estimation for the generalized Pareto distribution. Technometrics.

[CR16] Hosking JRM, Wallis JR, Wood EF (1985). Estimation of the generalized extreme-value distribution by the method of probability-weighted moments. Technometrics.

[CR17] Jenkinson AF (1955). The frequency distribution of the annual maximum (or minimum) values of metereological elements. Q J R Meteorol Soc.

[CR18] Katz RW (2010). Statistics of extremes in climate change. Clim Change.

[CR19] Leadbetter MR, Lindgren G, Rootzén H (1983). Extremes and related properties of random sequences and processes.

[CR20] Lye LM, Hapuarachchi KP, Ryan S (1993). Bayes estimation of the extreme-value reliability. IEEE Trans Reliab.

[CR21] Pickands J (1975). Statistical inference using extreme order statistics. Ann Stat.

[CR22] Pisarenko VF, Sornette D (2003). Characterization of the frequency of extreme earthquake events by the generalized Pareto distribution. Pure Appl Geophys.

[CR23] Prescott P, Walden AT (1980). Maximum likelihood estimation of the parameters of the generalized extreme-value distribution. Biometrika.

[CR24] Scarrott C, MacDonald A (2012). A review of extreme value threshold estimation and uncertainty quantification. REVSTAT.

[CR25] Smith RL, de Oliveira JT (1984). Threshold methods for sample extremes. Statistical extremes and applications.

[CR26] Smith RL (1985). Maximum likelihood estimation in a class of nonregular cases. Biometrika.

[CR27] VRA: Ghana’s Power Outlook. Technical report, Volta River Authority, Accra (2013). http://www.vra.com/resources/others/power_outlook_may_2014.pdf

